# Over-expression of Skp2 is associated with resistance to preoperative doxorubicin-based chemotherapy in primary breast cancer

**DOI:** 10.1186/bcr2122

**Published:** 2008-07-21

**Authors:** Shirly Davidovich, Ofer Ben-Izhak, Ma'anit Shapira, Boris Futerman, Dan D Hershko

**Affiliations:** 1 Department of Surgery A, Rambam Medical Center and the Technion-Israel Institute of Technology, 1 Efron Street, Haifa 31096, Israel; 2 Department of Pathology, Rambam Medical Center and the Technion-Israel Institute of Technology, 1 Efron Street, Haifa 31096, Israel; 3 Unit of Clinical Epidemiology, Rambam Medical Center and the Technion-Israel Institute of Technology, 1 Efron Street, Haifa 31096, Israel; 4 Breast Health Institute, Rambam Medical Center and the Technion-Israel Institute of Technology, 1 Efron Street, Haifa 31096, Israel

## Abstract

**Introduction:**

Preoperative chemotherapy is often used in patients with locally advanced breast cancer. However, commonly used clinical and pathological parameters are poor predictors of response to this type of therapy. Recent studies have suggested that altered regulation of the cell cycle in cancer may be involved in resistance to chemotherapy. Over-expression of the ubiquitin ligase Skp2 results in loss of the cell cycle inhibitor p27^Kip1 ^and is associated with poor prognosis in early breast cancer. The purpose of the present study was to examine the role of these proteins as predictors of clinical outcome and response to chemotherapy in locally advanced breast cancer.

**Methods:**

The expression levels of Skp2 and p27^Kip1 ^were determined by immunohistochemistry both before and after preoperative chemotherapy in 40 patients with locally advanced breast cancer. All patients were treated with cyclophosphamide/doxorubicin (adriamycin)/5-fluorouracil (CAF) and some patients received additional treatment with docetaxel. Expression data were compared with patients' clinical and pathological features, clinical outcome, and response to chemotherapy.

**Results:**

Skp2 expression before preoperative chemotherapy was inversely related to p27^Kip1 ^levels, tumor grade, and expression of estrogen and progesterone receptors. Both Skp2 and p27^Kip1 ^were found to be accurate prognostic markers for disease-free and overall survival. High preoperative expression of Skp2 was associated with resistance to CAF therapy in 94% of patients (*P *< 0.0001) but not with resistance to docetaxel.

**Conclusion:**

Skp2 expression may be a useful marker for predicting response to doxorubicin-based preoperative chemotherapy and clinical outcome in patients with locally advanced breast cancer.

## Introduction

Preoperative chemotherapy is widely used in the management of primary breast cancer, and particularly in patients who present with locally advanced disease [1-3]. Studies have clearly shown that initiating treatment with chemotherapy can lead to tumor regression in a substantial number of patients, thereby improving local control and allowing breast-conserving surgery in many patients without compromising clinical outcomes [3]. In addition, this practice permits direct assessment of tumor responsiveness to a given drug regimen and may allow one to determine the need to add or switch to a different regimen. However, not all tumors respond equally to a given chemotherapy combination. Some tumors may be responsive only to specific drugs, which may not be in the initial preoperative protocol used, whereas other tumors may not respond to a large variety of chemotherapies. This may lead to unnecessary exposure to drug side effects and loss of time to treatment, and may enable tumor progression. Unfortunately, commonly used clinical and pathological factors are poor predictors of response to chemotherapy. Therefore, identification of biological markers that can select those patients who are most likely to respond to specific preoperative chemotherapy is of the utmost importance.

The mechanisms responsible for resistance of cancer cells to chemotherapy, whether inherent or acquired, are poorly understood. Because cell cycle regulation is a key mechanism by which most chemotherapy agents exert their cytotoxic effect, alterations that may occur in cell cycle regulation in cancer may contribute to lack of response to some agents [4]. Under normal circumstances, progression of the cell cycle is tightly controlled by timely activation of cyclin-dependent kinases (Cdks) [5-7]. The progression of each phase of the cell cycle is promoted by phosphorylation of a variety of proteins by different members of the Cdk family, which in turn are dependent on the presence of a specific cyclin. In addition, these kinases are also negatively regulated by specific Cdk inhibitors at different phases in the cell cycle [7].

Recent studies have provided evidence that several Cdk inhibitors are targets for genetic changes or are disrupted by other oncogenic events in human cancers [8,9]. It was also suggested that some of these changes may have important impacts on the sensitivity of cancer cells to chemotherapy [10,11]. For example, p21^WAF1 ^(where the definition of WAF1 is 'wild-type p53 activated fragment 1') is a Cdk inhibitor that blocks the cell cycle at the G_1_/S transition by inhibiting the activity of Cdk2 and Cdk4/6 [7]. In response to DNA-damaging agents, wild-type 53 induces the expression of p21^WAF1^, leading to cell cycle arrest. Changes in the expression of p21^WAF1 ^were linked to drug sensitivity in various cancer cell lines. Cultured breast cancer cells deficient in p21^WAF1 ^exhibited increased response to DNA-damaging agents, whereas over-expression of p21^WAF1 ^in glioblastoma cells was associated with increased resistance to nitrosourea and cisplatin [10,12]. However, studies examining the significance of p21^WAF1 ^as a prognostic marker or as a predictor of chemoresistance in human patients yielded inconsistent findings; low levels of p21^WAF1 ^were independent prognostic markers for disease-free survival in some studies but not in others [13-15]. p21^WAF1 ^alone was not found to be associated with resistance to doxorubicin/mitomycin/5-fluorouracil or doxotaxel in patients with locally advanced breast cancer, but when combined with low murine double minute gene 2 (MDM2) protein levels it predicted good response to doxotaxel but not to mitomycin/5-fluorouracil [15].

Another Cdk inhibitor that plays a central role in cell cycle regulation and is thought to affect resistance to anti-cancer drugs is p27^Kip1^. p27^Kip1 ^is a member of the CIP/KIP (kinase inhibitor protein) family that negatively regulates protein kinases Cdk2/cylin E and Cdk2/cyclin A, which drive cells into the S-phase of the cell division cycle [7]. In contrast to p21^WAF1^, its role as a tumor suppressor and as a prognostic marker in human cancers is well established. Loss of p27^Kip1 ^contributes to uncontrolled tumor proliferation and is associated with high aggressiveness and poor prognosis in a large variety of cancers, including breast cancer [8,16,17]. It is now apparent that the loss of p27^Kip1 ^in human cancers results from increased protein degradation rather than from genetic mutations or decreased gene expression [18]. This increased degradation is mediated by the ubiquitin system, resulting in rapid proteasome-mediated degradation of p27^Kip1 ^[19]. The specificity of the ubiquitin system in targeting proteins for degradation is defined mainly by its ubiquitin ligase complexes [20]. The machinery that is involved in targeting p27^Kip1 ^for degradation is an SCF-type ubiquitin ligase complex that contains S-phase kinase protein 2 (Skp2) as the specific substrate-recognizing subunit [21,22].

The role of Skp2 as the main rate-limiting regulator p27^Kip1 ^degradation has been clearly demonstrated in both intact cells and in cell-free systems, as well as in many human cancers [21-25]. Skp2 was found to be an oncogene and an independent prognostic marker for disease-free and overall survival in breast cancer [26-28]. Moreover, recent studies have showed that Skp2 also regulates other cell cycle regulators that may contribute to cancer progression, including c-Myc, cyclin E, p57^Kip2^, p21^WAP1^, and E2F1 [29-32]. However, the effect of Skp2 on some of the latter proteins is complex. For example, it has been reported that Skp2 not only targets c-Myc for degradation, but also enhances its transcriptional activity. Thus, it may be expected that over-expression of Skp2 would decrease levels of c-Myc, whereas the growth-promoting action of c-Myc would actually increase [33].

Because alterations in the expression of p27^Kip1 ^profoundly affect cell cycle regulation in cancer, its role as a potential marker for chemoresistance has been examined in different cultured cancer cells. Poor response to cyclophosphamide was observed in cultured breast cancer cells expressing high levels of p27^Kip1^, but sensitivity was restored after downregulation of p27^Kip1 ^[10]. In contrast, low levels of p27^Kip1 ^were found to be involved in docetaxel resistance [34]. Other studies suggest that p27^Kip1 ^expression may have anti-apoptotic effect and prevent drug-induced apoptosis by DNA-damaging agents, such as cisplatin, leading to drug resistance [35]. Skp2 over-expression is the main factor involved in deregulation of p27^Kip1^, and Skp2 is also a regulator of other cell cycle proteins that are involved in tumor progression; therefore, it may also have important influence on drug resistance. Nevertheless, the role of Skp2 as a predictor to drug resistance remains unknown.

Doxorubicin (adriamycin) based chemotherapy is highly effective in the treatment of breast cancer and is among the more commonly used protocols for preoperative management of patients with locally advanced disease. However, despite its general efficiency, as many as 50% of patients do not respond adequately to this type of treatment. In the present study we examined the expression of p27^Kip1 ^and Skp2 before and after preoperative doxorubicin-based chemotherapy and analyzed their association with clinical and pathological parameters, including prognosis and response to chemotherapy.

## Materials and methods

### Patients

Forty patients with locally advanced primary breast cancer diagnosed and treated at Rambam Medical Center between 1999 and 2005 were included, once the approval of the institution's Human Investigation Committee had been obtained. All patients initially received the same preoperative chemotherapeutic regimen, which included cyclophosphamide 600 mg/m^2^, doxorubicin (adriamycin) 60 mg/m^2^, and 5-fluorouracil 600 mg/m^2^, once every 3 weeks for up to six cycles. Some patients received docetaxel (100 mg/m^2^, once every 3 weeks for up to four cycles) at the discretion of the treating medical oncologist. The median duration of treatment before surgery was 5 months (range 3 to 8 months). Additional postoperative treatment included radiotherapy and tamoxifen in estrogen receptor (ER) and/or progesterone receptor (PgR) positive patients. Response to chemotherapy was assessed in accordance with standard criteria as follows: complete pathological response was defined as the absence of malignant appearing cells in the surgical specimen; partial response was defined as at least 50% reduction in tumor size; and a reduction of less than 50% in tumor size was classified as stable disease (poor response). Therapy was to be terminated immediately if progressive disease became evident, and in such cases alternative chemotherapy or surgery was employed. Complete clinical and pathological data were available for all patients. Long-term follow-up data from the Medical Center's medical charts and the Israel Cancer Registry were assessed.

### Tissue specimen and immunohistochemistry

**Figure 1:**
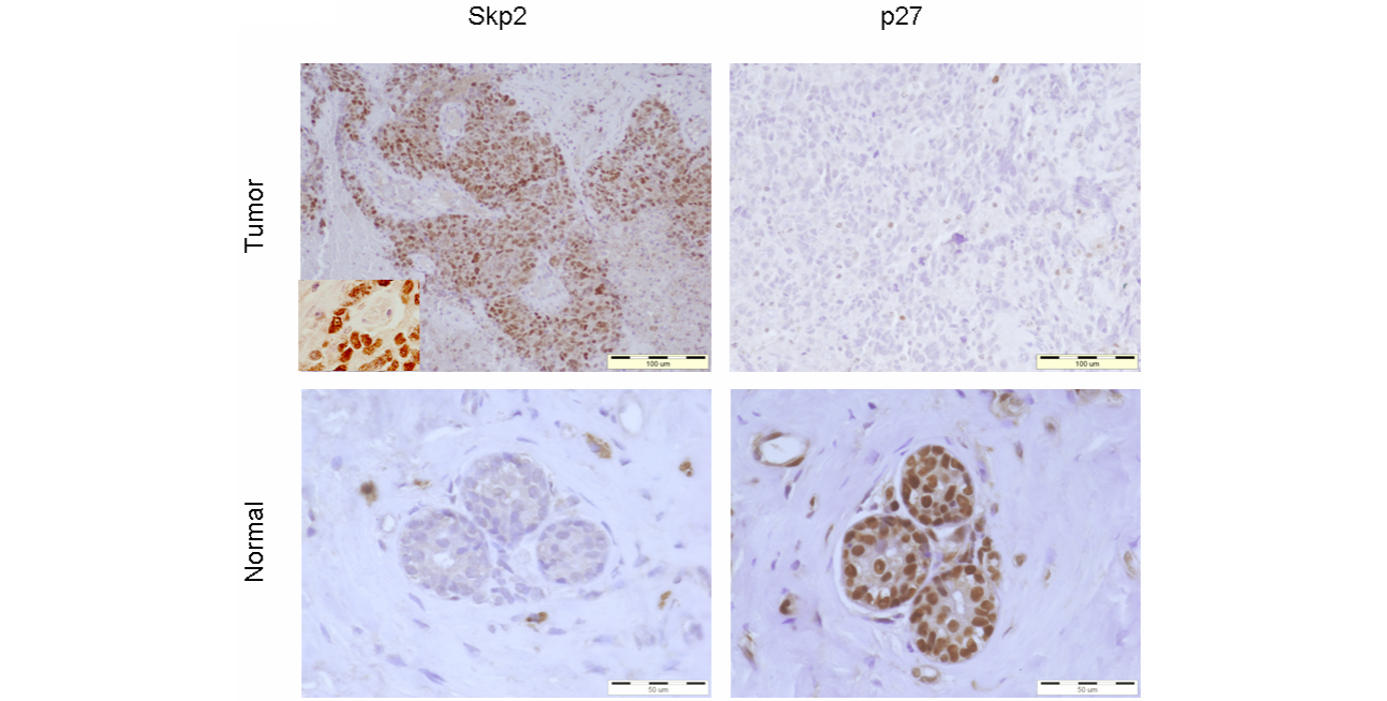
Representative immunohistochemistry slides for Skp2 and p27^Kip1 ^staining in locally advanced breast cancer. Tumor cells of pretreated grade II invasive duct cancer (T3N1MO) exhibiting strong and diffuse nuclear staining for Skp2 and low staining of p27^Kip1 ^(upper row and insert). Note the inversely low Skp2 staining followed by high p27^Kip1 ^staining in normal breast tissue (lower row). KIP, kinase inhibitor protein; Skp2, S-phase kinase protein 2.

Paired tissue specimen obtained by core biopsy before initiation of chemotherapy and from the surgical specimen after the completion of chemotherapy were examined. Immunohistochemical studies were performed on formalin-fixed, paraffin-embedded tissue sections [36]. Sections (5 μm) were deparaffinized with xylene and rehydrated in a series of ethanols. For epitope retrieval, slides were heated in 1 mmol/l EDTA buffer (pH 8), either in microwave oven at 92°C for 20 minutes for p27^Kip1^, or in an Antigen Retrieval Processor (Milestone Inc., Sorsiole, Italy) at 120°C for 8 minutes for Skp2. After cooling, slides were washed in distilled water. Skp2 staining was carried out in the NexES IHC Immunostainer (Ventana Medical Systems, Tucson, AZ, USA), in accordance with the manufacturer's instructions, using a monoclonal antibody (Zymed Inc., San Francisco, CA, USA) diluted at 1:100. This antibody cannot distinguish between Skp2 and the F-box protein Skp2B. Skp2B has little if any influence on the degradation of p27, but in contrast to Skp2 (which is strictly localized in the nucleus), Skp2B is localized only in the cytoplasm [37,38]. Because of the restriction of our analysis to expression of nuclear Skp2, the results reflect only levels of Skp2, and not those of Skp2B (Figure 1).

Slides for p27^Kip1 ^staining were treated for 10 minutes with 3% H_2_O_2 _in methanol in order to block endogenous peroxidase, and for 30 minutes with 10% nonimmune rabbit serum to block non specific protein binding. The slides were then washed in water and soaked in washing buffer (pH 7.4; Optimax, Biogenex, San Ramon, CA, USA) for 5 minutes. For p27^Kip1 ^staining, slides were incubated overnight at 4°C with the monoclonal p27^Kip1 ^antibody diluted at 1:500. Staining was completed with Histostain-plus kit (Zymed Inc.), in accordance with the manufacturer's instructions. Color reaction product was developed with amino ethyl carbazole. All sections were counterstained with hematoxylin. For negative controls, slide sections that were positive for staining were treated with 10% nonimmune rabbit serum (Zymed Inc.) instead of the primary antibody. No staining was observed in any of these controls.

Scoring of immunohistochemical slides was done according to the percentage of tumor cells exhibiting nuclear staining. The range of Skp2 expression was between 0% and 40% of cells, and that of p27 was between 0% and 80%, similar to values reported in previous studies [16-18,26,36]. To define high and low protein expression, we used a cut-off of 50% for p27^Kip1 ^and 10% for Skp2, which is the cut-off used in the above-mentioned studies because it correlated well with quantitative immunoblot data. These values were determined following analysis of continuous variables (increments of 10%) that showed that within the group of high expression no significant changes were found. When stained, cells exhibited similar intensity of nuclear staining of Skp2 regardless of the percentage of cells stained, and therefore the intensity of staining was not included in the score. The specificity of immunohistochemistry staining procedures for p27^Kip1 ^and Skp2 was previously verified by comparing the protein levels, as determined by immunohistochemistry, with the protein levels determined by immunoblot analysis from the same tumor specimens [23]. The examining pathologist was blinded to the patients' clinical outcome and response rate of response to chemotherapy

### Statistical analysis

Statistical data analyses were performed using SPSS 11.0 statistical software package (SPSS Inc., Chicago, IL, USA). First, the relationship between p27^Kip1 ^and Skp2 and between protein levels and different clinical and pathological features and response to chemotherapy were explored using cross tabulation and Pearson's χ^2^. Survival curves were constructed using the Kaplan-Meier method and multivariate analysis by Cox regression; *P *values less than 0.05 were considered statistically significant.

## Results

### Skp2 expression before preoperative chemotherapy is inversely related to p27^Kip1 ^levels, tumor differentiation, ER/PgR expression, and prognosis in locally advanced breast cancer

Initially, we examined the expression of Skp2 and p27^Kip1 ^in 40 tumor samples obtained from patients with locally advanced breast cancer before the initiation of preoperative chemotherapy. The clinical and pathological characteristics of these patients are summarized in Table 1. Skp2 levels were high in 17 patients (42%) and p27^Kip1 ^levels were high in 32 patients (80%). Skp2 levels were inversely related to p27^Kip1 ^levels in 70% of tumors (29 patients; *P *= 0.011). When Skp2 levels were low, p27^Kip1 ^levels were high in 95.7% of patients, but in those tumors in which Skp2 levels were high p27^Kip1 ^levels were low in only 41% of patients. Ten patients had high Skp2/high p27^Kip1 ^levels and one patient had low Skp2/low p27^Kip1 ^levels. A typical representative immunohistochemical sample is shown in Figure 1.

**Table 1:** Clinical and pathological characteristics of patients

Characteristic	Value
Age (years; median [range])	47 (29 to 79)
Histology (*n *[%])	
Ductal	33 (82%)
Lobular	7 (18%)
Tumour grade (*n *[%])	
G1	3 (7%)
G2	21 (53%)
G3	16 (40%)
Primary tumor (*n *[%])	
T2	7 (17%)
T3	20 (50%)
T4	13 (33%)
Lymph node status (*n *[%])	
Positive	24 (60%)
Negative	16 (40%)
Primary tumor (*n *[%])	
IIA	4 (10%)
IIB	8 (20%)
IIIA	15 (33%)
IIIB	18 (38%)
Estrogen receptor status (*n *[%])	
Positive	24 (60%)
Negative	16 (40%
Progesterone receptor status (*n *[%])	
Positive	22 (55%)
Negative	18 (45%)
Her2 receptor status (*n *[%])	
Positive	9 (23%)
Negative	31 (77%)

To examine the relationship between the expression of these proteins and common parameters associated with tumor behavior, we compared Skp2 and p27^Kip1 ^levels with the clinicopathological features described in Table 1. A significant inverse correlation was found between Skp2 expression and tumor differentiation (*P *= 0.001), ER expression (*P *= 0.006), and PgR expression (*P *= 0.005; Table 2). Thus, high expression of Skp2 was associated with loss of tumor differentiation and negative ER or PR expression. We did not observe a significant correlation between Skp2 levels and patient age (*P *= 0.083), lymph node status (*P *= 0.083), tumor size (*P *= 0.063), disease stage (*P *= 0.449), or Her2/neu expression (*P *= 0.088; Table 2). Examination of the relation between p27^Kip1 ^and patients' clinicopathological characteristics revealed a strong positive association between the expression of p27^Kip1^, and ER expression (*P *= 0.001) and PR expression (*P *= 0.017), and an inverse association with Her2/neu expression (*P *= 0.022), but not with the other clinicopathological parameters (Table 2).

**Table 2:** Expression of Skp2 and p27^Kip1 ^in relation to the clinical and pathological characteristics of patients

Characeristic	Skp-2			p27^kip1^		
	
	High	Low	*P *value	High	Low	*P *value
Age (years)						
>50	8	17	0.083	20	5	0.591
<50	9	16		13	2	
Tumor grade						
G1	3	0	0.001	3	0	0.162
G2	4	19		20	2	
G3	13	3		11	5	
Primary tumor						
T2	1	6	0.063	7	0	0.105
T3	12	8		14	6	
T4	4	9		12	1	
Lymph node status						
Positive	8	17	0.083	21	4	0.747
Negative	9	6		12	3	
Stage						
IIA	1	3	0.449	4	0	0.407
IIB	5	3		6	2	
IIIA	7	8		11	4	
IIIB	4	9		12	1	
Estrogen receptor status						
Positive	6	18	0.006	24	0	0.001
Negative	11	5		9	7	
Progesterone receptor status						
Positive	5	17	0.005	21	1	0.017
Negative	12	6		12	6	
Her2 receptor status						
Positive	6	3	0.088	3	5	0.002
Negative	11	20		30	2	

*P *values relate to the differences between protein expression and the variables. *P *value < 0.05 was considered statistically significant. T, tumor size (according to the American Joint Committee on Cancer TNM staging system). KIP, kinase inhibitor protein; Skp2, S-phase kinase protein 2.

**Figure 2:**
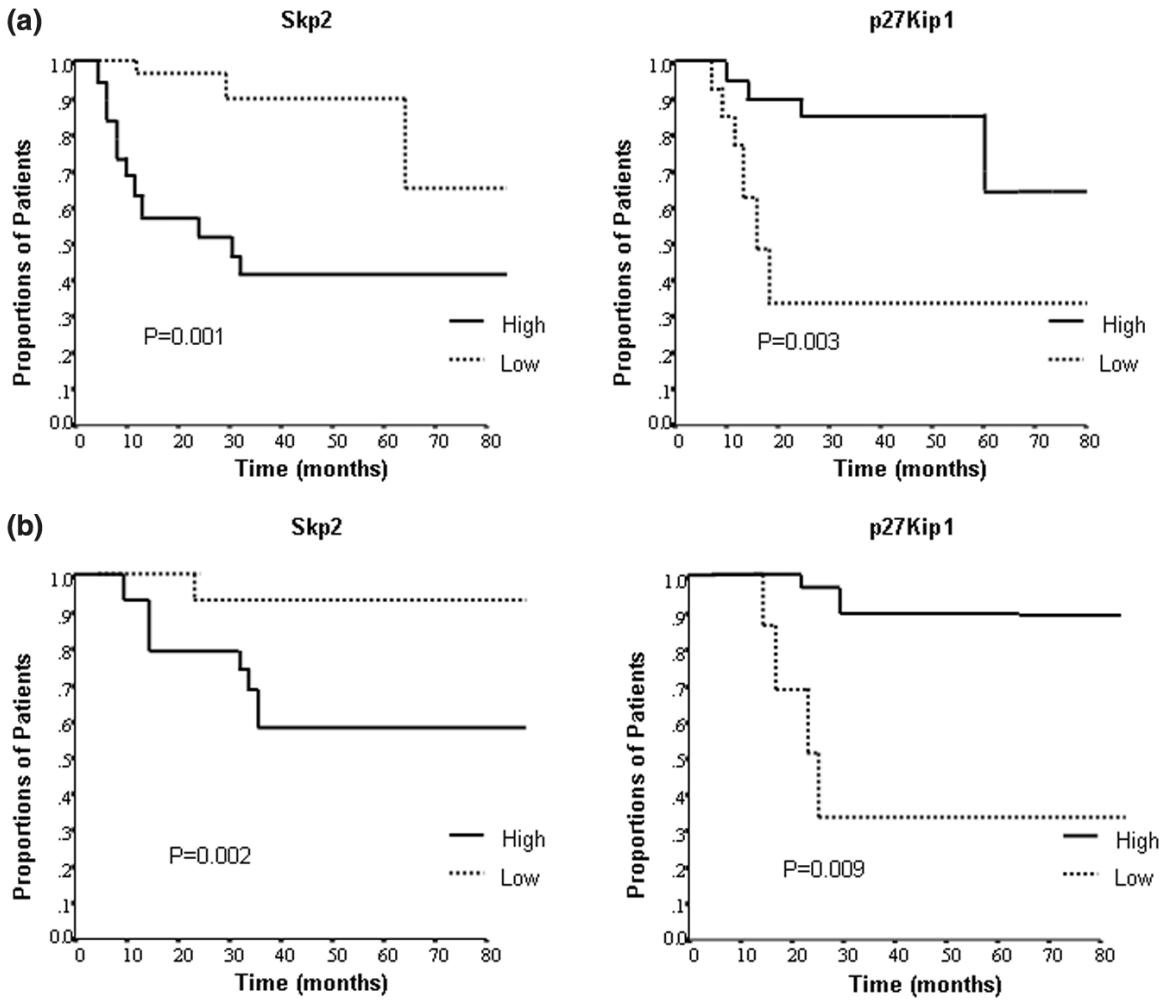
Pretreatment Skp2 and p27^Kip1 ^expression levels and survival. Shown are the associations between pretreatment Skp2 or p27^Kip1 ^expression and **(a) **disease-free survival and **(b) **overall survival rates. Analyses were constructed using the Kaplan-Meier method. KIP, kinase inhibitor protein; Skp2, S-phase kinase protein 2.

With a mean follow up of 42 months (18 to 82 months), disease recurrence was observed in 12 patients (30%). The mean ± standard deviation time to recurrence was 21 ± 4.6 months, and overall seven patients died from their disease. We found that the expression of Skp2 and p27^Kip1 ^(pre-chemotherapy) was highly predictive for disease-free and overall survival (Figure 2). Thus, patients with high Skp2 expression had significantly poorer disease-free and overall survival than did patients expressing low Skp2 levels (*P *= 0.001 and *P *= 0.002, respectively). Similarly, patients exhibiting low p27^Kip1 ^levels had significantly poorer survival rates than did patients expressing high p27^Kip1 ^levels (*P *= 0.003 and *P *= 0.009, respectively). Among the clinical parameters, young age, negative ER expression, and lack of response to cyclophosphamide/doxorubicin (adriamycin)/5-fluorouracil (CAF) were significantly associated with shorter disease-free survival. Multivariate analysis of all pretreatment variables, including Skp2 expression, p27^Kip1 ^expression, age, tumor size, tumor grade, nodal status, and ER, PR and Her2/neu receptor expressions, showed that Skp2 expression (*P *= 0.002; odds ratio = 8.91) and young age (*P *= 0.034) were the strongest predictors for poor disease-free survival.

To examine whether chemotherapy affected the expression of Skp2 and p27^Kip1^, we analyzed the expression of these proteins after the completion of chemotherapy. Analysis was performed on 35 tissue specimen (five patients had complete pathological response). Overall, there were no significant changes in the expression of either Skp2 or p27^Kip1 ^after chemotherapy (*P *= 0.190 and *P *= 0.104, respectively). However, in patients presenting with high Skp2, the expression of Skp2 was downregulated after chemotherapy in 60% of tumors (9/15), but expression was not upregulated in any of the tumors with initially low levels of Skp2 (*P *= 0.001). Surprisingly, despite the decrease in Skp2 levels after chemotherapy, p27^Kip1 ^levels were not increased in any of these tumors. Moreover, p27^Kip1 ^expression was in fact downregulated by chemotherapy in 24% of tumors (7/29 patients), and in four patients both Skp2 and p27^Kip1 ^levels were downregulated.

### Skp2 over-expression is associated with resistance to CAF but not to docetaxel

We next examined the association between pretreatment expression levels of Skp2 and p27^Kip1 ^and resistance to chemotherapy. All patients in this study were initially treated with doxorubicin-based chemotherapy (CAF). A decrease of at least 50% in tumor size was defined as a partial response, whereas a reduction of less than 50% in tumor size was classified as stable disease or poor response. The associations of expression levels of Skp2 and p27^Kip1^, and patients' clinical and pathological characteristics with response to CAF are outlined in Table 3. Twenty patients (50%) exhibited poor or no response to CAF. Skp2 was over-expressed in 16 (80%) of these patients. Only one case was detected in which Skp2 over-expression was associated with partial response to CAF. Thus, high Skp2 levels were associated with poor response to chemotherapy in 94.1% of cases in this study (*P *< 0.0001). On the other hand, low Skp2 accurately predicted response to CAF in 79.2% of cases (19/24 patients). p27^Kip1 ^expression was also found to be a predictor for response, but to a lesser extent than Skp2 (*P *= 0.037). p27^Kip1 ^expression was high in 19 (95%) of the tumors responding to CAF. However, p27^Kip1 ^expression was also high in 14 (70%) of the poor responders. Thus, high levels of p27^Kip1 ^accurately predicted response to CAF in only 57.6% of cases in this study. In contrast, low expression of p27^Kip1 ^was associated with response to CAF in one patient only, thus correlating with poor response to CAF in 85.7% of cases. We also examined the correlation between the patients' clinical and pathological characteristics and response to CAF. Poor tumor grade (*P *= 0.012) and clinically negative lymph nodes (*P *= 0.022) were the only parameters found to be associated with poor response to CAF (Table 3). Multivariate analysis revealed that Skp2 was the strongest predictor for response to CAF (*P *> 0.001; ods ratio = 13.73).

**Table 3:** The association of response to preoperative CAF treatment and patients' clinical and pathological characteristics with expression levels of Skp2 and p27^Kip1^

Characteristics	Response		*P *value
		
	+	-	
Age (years)			
>50	8	17	0.13
<50	9	6	0
Tumor grade			
G1 + G2	16	8	
G3	4	12	0.010*
Primary tumor			
T2	5	2	0.151
T3	7	13	
T4	8	5	
Lymph node status			
Positive	16	9	0.022*
Negative	4	11	
Stage			
IIA	3	1	0.289
IIB	2	6	
IIIA	7	8	
IIIB	8	5	
Estrogen receptor status			
Positive	15	9	0.053
Negative	5	11	
Progesterone receptor status			
Positive	14	8	0.057
Negative	6	12	
HER-2 receptor status			
Positive	6	3	0.212
Negative	14	17	
p27^Kip1^			
High	19	16	0.037*
Low	1	4	
Skp2			
High	1	16	0.0001*
Low	19	4	

*P value < 0.05 was considered statistically significant. +, ≥ 50% reduction in tumor size; -, no response or <50% reduction in tumor size; KIP, kinase inhibitor protein; Skp2, S-phase kinase protein 2.

Nineteen patients (47.5%) also received docetaxel after CAF treatment. In 89.4% of these patients the reason for adding or switching to docetaxel was poor response to CAF. Partial or complete (2 patients) response was observed in 10 of these patients (47.3%). Twelve patients (63.1%) had high Skp2 levels and 14 patients (73.6%) had high p27^Kip1 ^levels. We did not find a correlation between the expression of either Skp2 or p27^Kip1 ^and the rate of response to docetaxel (*P *= 0.764 and *P *= 0.701, respectively). Of clinical importance, however, is the observation that 50% of the high-Skp2 tumors that did not respond to CAF exhibited a good response to docetaxel, suggesting that docetaxel may be a better initial choice than CAF in this subset of patients.

Complete tumor pathological response was identified in five patients. A correlation between the expression of the proteins and this type of response could not be established, mainly because of the small number of patients in this subgroup. Similarly, there was no correlation between complete elimination of nodal metastases by chemotherapy (four patients) and Skp2 or p27^Kip1 ^expression (*P *= 0.674 and *P *= 0.711, respectively).

## Discussion

The use of preoperative chemotherapy has become the standard of care in patients with locally advanced breast cancer. However, the correlation between commonly used clinical and pathological features and respond to various chemotherapy regimens is poor. Adriamycin-based chemotherapy is commonly used as the first-line treatment in this clinical setting. However, adriamycin has considerable toxic effects and, in particular, cardiac toxicity. This clinical side effect may be exacerbated in patients with positive Her2/neu receptor status by co-treatment with trastuzumab. Thus, the search for specific molecular markers that may serve as predictors for response is of considerable clinical importance.

The expression of various cell cycle regulators was found to be accurate in predicting tumor biology and clinical outcome. Some of these proteins, including p27^Kip1^, were also found to affect response to chemotherapy in different cancers and cancer cell lines. However, the data available on the impact of p27^Kip1 ^and other cell cycle proteins as predictors to response to preoperative chemotherapy in patients with locally advanced breast cancer are limited. In a recent report, Pohl and coworkers [40] examined the expression of various cell cycle regulatory proteins, including p27^Kip1^, in locally advanced breast cancer and their association with complete pathological response to preoperative chemotherapy. Twenty nine patients were treated with CAF and 36 patients with epirubicin/docetaxel. High expression of Ki-67 was associated with complete pathological response, but there was no correlation of expressions of p53, p21^WAF1^, p27^Kip1^, and cyclin D with complete response to chemotherapy. However, that study did not address the issue of whether any of these proteins was associated with partial response, defined as a reduction of at least 50% in tumor size. Because one of the main aims of using preoperative chemotherapy is to achieve significant reduction in tumor size in order to allow good local control and possibly even permit breast-conserving surgery, this issue has important clinical implications. Moreover, in as many as 60% of complete clinical response, residual tumor is found on pathological analysis. Thus, at present all patients receiving preoperative chemotherapy must also undergo surgery, so a marker that can predict good response rather than complete pathological response may be of great clinical importance. Ideally, such a marker should be reliably reproduced, rapidly performed and at minimal cost, such as immunohistochemical studies. Studies have shown that gene expression profiling studies may be used as predictors of response to chemotherapy, but these studies are time consuming and expensive [41,42].

The role of Skp2 as an oncogene responsible for downregulation of p27^Kip ^protein levels is well established in a wide variety of cancers, including early breast cancer. Studies have repeatedly shown that Skp2 enhances tumor progression and is independently associated with poor prognosis, suggesting that it may be a novel potential target for intervention. In the present study we examined the expression of p27^Kip1 ^and Skp2 in patients with locally advanced breast cancers, before and after chemotherapy, in order to determine their potential roles as prognostic factors and as predictors of response to preoperative chemotherapy. Our findings suggest that these proteins, and in particular Skp2, may have important benefits in this regard.

First, similar to previous reports in early breast cancer, in locally advanced disease we found that high expression of Skp2 or low expression of p27^Kip1 ^correlated strongly with pathological features associated with aggressive tumors, including poor tumor differentiation and lack of receptors to estrogen and progesterone. In addition, we found both Skp2 and p27^Kip1 ^to be accurate predictors of disease-free and overall survival, which suggests that these proteins may also be useful markers in locally advanced cancer. Patients with locally advanced disease already present with clinical features that are strongly associated with poor prognosis, such as large tumor size, positive lymph nodes and advanced stage. Therefore, these features may not provide additional prognostic information within this group of patients, as compared with molecular markers such as Skp2, which may provide additional and important information in this subset of patients. Interestingly, the percentage of patients presenting with high Skp2 levels in locally advance disease was similar to that of patients presenting with high Skp2 levels in early breast cancer (40% and 42%, respectively) [36]. This suggests that high expression of Skp2 in a given tumor is an inherent feature of the tumor's biology rather than one that is acquired during progression of the disease. Second, we found that doxorubicin-based chemotherapy did not significantly alter Skp2 or p27^Kip1 ^levels, but when Skp2 levels did decrease no apparent changes were observed in p27^Kip1 ^levels. The mechanism underlying these observations is unclear at present. However, we previously examined the effect of doxorubicin on Skp2 expression in various breast cancer cell lines. We found that although Skp2 levels decreased in some cell lines, in others they did not, and these changes were dependent on the specific cell-type effect of doxorubicin on cell cycle arrest [43]. Moreover, in these experiments changes in Skp2 levels also did not translate into changes in p27^Kip1 ^levels. It is noteworthy, however, that despite the lack of changes in p27^Kip1^, Skp2 levels were independently associated with a significantly better disease-free survival. This supports the concept that Skp2 has other important oncogenic effects and that reduction in Skp2 levels is a rational therapeutic objective.

Finally, we found that Skp2 is an accurate predictor of response to doxorubicin-based chemotherapy. Skp2 levels were high in 80% of the poor responders; more specifically, when Skp2 levels were high, 94% of the patients did not respond sufficiently to doxorubicin-based therapy. Multivariate analysis, including all available clinical and pathological parameters, revealed that Skp2 is an extremely accurate predictor for response to doxorubicin-based (*P *> 0.001; odds ratio = 13.73). Although the statistical significance for the ability of Skp2 to predict response to CAF is high, the number of patients in this study is rather small. Therefore, confirmatory evidence from larger studies is required before we may propose that this marker be used to decide whether to administer doxorubicin-based preoperative chemotherapy to patients with breast cancer. It is also important to bear in mind that two additional drugs are included in this regimen, which might have influenced the findings of the present study to some degree. In contrast, the relationship between Skp2 expression and docetaxel resistance is difficult to interpret. The patients who received docetaxel were a selected and relatively small group of patients who did not respond to anthracycline-based chemotherapy. Although in this group of patients the expression of Skp2 was not associated with docetaxel resistance, larger studies conducted in patients receiving taxane-based regimens are needed to determine the role of Skp2 as a predictor in taxane-based chemotherapy.

## Conclusion

The results of the present study suggest that Skp2 may be an accurate biological marker for prognosis as well as a predictor of response to doxorubicin-based chemotherapy in locally advanced breast cancer. Furthermore, these results support previous studies that emphasize the oncogenic properties of Skp2; hence, this protein may be a novel target for future interventions.

## Abbreviations

CAF = cyclophosphamide/doxorubicin (adriamycin)/5-fluorouracil; Cdk = cyclin-dependent kinase; ER = estrogen receptor; KIP = kinase inhibitor protein; PgR = progesterone receptor; Skp2 = S-phase kinase protein 2; WAF1 = wild-type p53 activated fragment 1.

## Competing interests

The authors declare that they have no competing interests.

## Authors' contributions

SD carried out the immunohistochemical studies, performed the statistical analysis and participated in drafting the manuscript. OBI carried out the pathological analysis and scoring of the immunohistochemical results. MS carried out part of the immunohistochemical studies and study design. BS performed the statistical analysis. DDH conceived of the study, participated in the design of the study, participated in the sequence alliance and helped to draft the manuscript. All authors read and approved the final version.
